# Supplementary material for: MultiSCRIPT-Cycle 1—a pragmatic trial embedded within the Swiss Multiple Sclerosis Cohort (SMSC) on neurofilament light chain monitoring to inform personalized treatment decisions in multiple sclerosis: a study protocol for a randomized clinical trial
Source: Trials. 2024 Sep 11;25:607. doi: 10.1186/s13063-024-08454-6 (PMC11391827; doi:10.1186/s13063-024-08454-6)
Supplement: Supplementary file 2 — Supplementary Material 2. [file 13063_2024_8454_MOESM2_ESM.docx]

**Appendix 1: PRECIS-2 assessment**

PRECIS-2 scoring results are (pragmatic attitudes are reflected by a maximum of 5 points, while explanatory characteristics by lower scores) - Eligibility 5 (broad eligibility criteria with only one exclusion criterion); Recruitment 5 (patients will be recruited during their usual care yearly visit); Setting 5 (embedded in the SMSC usual care and 7 centers from the cohort will be recruiting); Organization 5 (make use of the already existing SMSC organization in place); Flexibility – delivery 5 (treatment guidelines pre-specified but as any guidelines, it will be the physicians and patients’ decision to follow them); Flexibility – Adherence 5 (no mitigation measures will be taken to increase the use of diagnostic information); Follow-up 5 (number of visits and routine assessment increased in intervention group, but part of the biomarker monitoring intervention; no change in control group); Primary Outcome 5 (decision-relevant and patient reported outcome); Primary analysis 5 (intention to treat).
