# Supplementary material for: MultiSCRIPT-Cycle 1—a pragmatic trial embedded within the Swiss Multiple Sclerosis Cohort (SMSC) on neurofilament light chain monitoring to inform personalized treatment decisions in multiple sclerosis: a study protocol for a randomized clinical trial
Source: Trials. 2024 Sep 11;25:607. doi: 10.1186/s13063-024-08454-6 (PMC11391827; doi:10.1186/s13063-024-08454-6)
Supplement: Supplementary file 3 — Supplementary Material 3. [file 13063_2024_8454_MOESM3_ESM.docx]

**Appendix 2: Treatment decision algorithms**

The following treatment decision algorithms are intended to serve as treatment decision aids for the clinical application of serum neurofilament light chain values within MultiSCRIPT-Cycle 1 to guide escalation and de-escalation of disease modifying therapies (DMTs) in patient with relapse-remitting multiple sclerosis (RRMS). They represent a minimal set of treatment decision algorithms that experts have reach a certain level of agreement on.

The proposed treatment decision algorithms are not meant to cover all unique cases that physicians might encounter in the clinics. We acknowledge that treatment modifications related to pregnancies, treatment intolerances, comorbidities and other causes may not be captured here. The proposed algorithms are not binding and do not intend to supplant patients' and physicians' preferences. Physician at their discretion may pursue any *additional clinical and/or clinical assessment* as per usual care to inform the decision.

The treatment decision algorithms rely on the following definitions:

**Usual care arm:** Consider escalation, if there is evidence of disease activity (clinically or MRI-activity)

**NEDA:** no evidence of disease activity

**NEDA2:** no relapse, no EDSS worsening

**NEDA3:** no relapse, no EDSS worsening, no MRI activity

**EDSS worsening:**defined as an increase of ≥1.5 points from an EDSS of 0, ≥1.0 point from an EDSS of 1.0-5.0 or ≥0.5 point from an EDSS ≥5.5

**MRI activity:** Any unequivocal new or enlarging T2w lesion (***after adequate re-baselining***) or contrast enhancement on T1w images on brain or spinal cord MRI according to the consensus between local neuroradiologist and treating neurologist.

**Evidence of disease activity based on sNfL** is defined using sNfL>90th percentile ([Benkert et al. Lancet Neurology 2022](https://pubmed.ncbi.nlm.nih.gov/35182510/)) and ***once other potential causes of high sNfL have been excluded***(e.g. trauma, stroke, relevant sports-related head injury, at least medium severe renal failure (GFR < 60 mL/min/1.73 m2), suboptimally treated diabetes mellitus or any other concomitant disease that may lead to relevant neuroaxonal damage)

| **DMT CLASSIFICATION** | **Delphi results** |
| --- | --- |
|  |  |
| **Low efficacy:** glatiramer acetate – interferon beta – teriflunomide | 97% Strong consensus |
|  |  |
| **Medium efficacy:** fumarates – S1P modulators – cladribine | 83% Broad consensus |
|  |  |
| **High efficacy:** alemtuzumab – anti B cell therapy – natalizumab | 100% Full agreement |

| **ESCALATION TREATMENT DECISION ALGORITHMS** | **Delphi results** |
| --- | --- |
|  |  |
| **TREATMENT INITIATION FROM UNTREATED** | |
| If your patient is currently **untreated** and has high sNfL (>90th percentile) jointly consider with your patient to initiate DMT if your patient has…. | |
| - **NEDA2** plus **MRI activity with at least 2** new/enlarging T2w lesions or contrast enhancing T1w lesions | 97% Strong consensus |
| - **NEDA2** plus **MRI activity with at least 1** new/enlarging T2w lesion or contrast enhancing T1w lesion | 93% Strong consensus |
| - **NEDA 3** | 80% Broad consensus |
|  |  |
| **ESCALATION FROM LOW DMT** | |
| If your patient is currently receiving a **low efficacy DMT** for at least 9 months and has high sNfL (>90thpercentile) jointly consider with your patient to escalate to medium or high efficacy DMT if your patient has….. | |
| - **NEDA2** plus **MRI activity with at least 2** unequivocal new/enlarging T2w lesions or contrast enhancing T1w lesions | 97% Strong consensus |
| - **NEDA2** plus **MRI activity with at least 1** unequivocal new/enlarging T2w lesion or contrast enhancing T1w lesion | 97% Strong consensus |
| - **NEDA3** | 67% Moderate consensus |
|  |  |
| **ESCALATION FROM MEDIUM DMT** | |
| If your patient is currently receiving a **medium efficacy DMT** for at least 9 months and has high sNfL (>90thpercentile) jointly consider with your patient to escalate to high efficacy DMT if your patient has… | |
| - **NEDA2** plus **MRI activity with at least 2** unequivocal new/enlarging T2w lesions or contrast enhancing T1w lesions | 100% Full agreement |
| - **NEDA2** plus **MRI activity with at least 1** unequivocal new/enlarging T2w lesion or contrast enhancing T1w lesion | 93% Broad consensus |
| - **NEDA3** | 67% Moderate consensus |

| **HORIZONTAL SWITCH TREATMENT DECISION ALGORITHMS** | **Delphi results** |
| --- | --- |
|  |  |
| **HORIZONTAL SWITCH FROM NATALIZUMAB** | |
| If your patient is currently receiving **natalizumab** and has high sNfL (>90thpercentile) jointly consider with your patient to switch to a different mode of action high efficacy DMT if your patient has… | |
| - **at least 9 months** natalizumab plus **NEDA2** plus **MRI activity with at least 2** unequivocal new/enlarging T2w lesions or contrast enhancing T1w lesions | 97% Strong consensus |
| - **at least 9 months** natalizumab plus **NEDA2** plus **MRI activity with at least 1** unequivocal new/enlarging T2w lesion or contrast enhancing T1w lesion | 84% Broad consensus |
| - **at least 12 months** natalizumab plus **NEDA3** | 54% Moderate consensus |
|  |  |
| **HORIZONTAL SWITCH FROM ANTI-B CELL THERAPY** | |
| If your patient is currently receiving **anti-B cell therapy** and has high sNfL (>90thpercentile) jointly consider with your patient to switch to a different mode of action high efficacy DMT if your patient…. | |
| - **at least 9 months** anti-B cell therapy plus **NEDA2** plus **MRI activity with at least 2** unequivocal new/enlarging T2w lesions or contrast enhancing T1w lesions | 93% Broad consensus |
| - **at least 9 months** anti-B cell therapy plus **MRI activity** plus **confirmed EDSS worsening or relapse** | 93% Broad consensus |
| - **at least 12 months** anti-B cell therapy plus **NEDA2** plus **MRI activity with at least 1** unequivocal new/enlarging T2w lesion or contrast enhancing T1w lesion | 92% Broad consensus |
| - **at least 12 months** anti-B cell plus **no MRI activity but relapse** | 83% Broad consensus |
| - **at least 12 months** anti-B cell plus **no MRI activity but confirmed EDSS worsening** | 71% Moderate consensus |
|  |  |
| **HORIZONTAL SWITCH FROM CLADRIBINE** | |
| If your patient is received the second cycle **cladribine** and has high sNfL (>90thpercentile) jointly consider with your patient to switch to a different mode of action or regimen strategy of DMT if your patient has…. | |
| - **at least 6 months** after the second cycle plus **NEDA2** plus **MRI activity with at least 2** unequivocal new/enlarging T2w lesions or contrast enhancing T1w lesions | 97% Strong consensus |
| - **at least 12 months** after the second cycle plus **NEDA3** | 83% Broad consensus |
| - **at least 6 months** after the second cycle plus **NEDA2** plus **MRI activity with at least 1** unequivocal new/enlarging T2w lesion or contrast enhancing T1w lesion | 81% Broad consensus |

| **DE-ESCALATION TREATMENT DECISION ALGORITHMS** | **Delphi results** |
| --- | --- |
|  |  |
| **DE-ESCALATION FROM ANTI-B CELL THERAPY** | |
| If your patient is currently receiving **anti-B cell therapy** for at ***least 2 years***, has **NEDA3 for the past 2 years** and has normal sNfL (< 80th percentile) jointly consider with your patient to perform 6-monthly cMRI and 6-monthly sNfL measurement and de-escalate by **extending treatment interval up to 12 months (Ocrevus, Rituximab) or 8 weeks (Kesimpta)** | 74% Moderate consensus |
|  |  |
| **DE-ESCALATION FROM FUMARATES** | |
| If your patient **is >60 years old**, currently receiving **fumarates** for at **least 5 years**, has **NEDA3 for the past 5 years**, **pre-treatment activity was low** (e.g., less than 1 relapse per year) and has normal sNfL (<80th percentile) jointly consider with your patient to perform 6-monthly cMRI and 6-monthly sNfL measurement and **stopping fumarates** | 74% Moderate consensus |
|  |  |
| **DE-ESCALATION FROM LOW EFFICACY DMT** | |
| If the patient wishes to stop treatment and is **>60 years old**, currently receiving **low efficacy DMT** for at **least 5 years**, has **NEDA3 for the past 5 years** and has normal sNfL (< 80th percentile) jointly consider with your patient to perform 6-monthlycMRI and 6-monthly sNfL measurement and **stopping DMT treatment** | 87% Broad consensus |
